# DC-based smart PV-powered home energy management system based on voltage matching and RF module

**DOI:** 10.1371/journal.pone.0185012

**Published:** 2017-09-21

**Authors:** Ahmad H. Sabry, W. Z. W. Hasan, MZA Ab. Kadir, M. A. M. Radzi, S. Shafie

**Affiliations:** 1 Control and Automation, Faculty of Engineering, UPM, Serdang, Malaysia; 2 Faculty of Engineering, UPM, Serdang, Malaysia; Chongqing University, CHINA

## Abstract

The main tool for measuring system efficiency in homes and offices is the energy monitoring of the household appliances’ consumption. With the help of GUI through a PC or smart phone, there are various applications that can be developed for energy saving. This work describes the design and prototype implementation of a wireless PV-powered home energy management system under a DC-distribution environment, which allows remote monitoring of appliances’ energy consumptions and power rate quality. The system can be managed by a central computer, which obtains the energy data based on XBee RF modules that access the sensor measurements of system components. The proposed integrated prototype framework is characterized by low power consumption due to the lack of components and consists of three layers: XBee-based circuit for processing and communication architecture, solar charge controller, and solar-battery-load matching layers. Six precise analogue channels for data monitoring are considered to cover the energy measurements. Voltage, current and temperature analogue signals were accessed directly from the remote XBee node to be sent in real time with a sampling frequency of 11–123 Hz to capture the possible surge power. The performance shows that the developed prototype proves the DC voltage matching concept and is able to provide accurate and precise results.

## Introduction

The need for new technologies to manage the energy of household appliances is growing in proportion to the increase in smart homes and the individual's standard of living. An embedded system with minimum wiring and consumption becomes one of the objectives of advanced home power management. For solar-powered systems in the residential sector, Wireless Sensor Network (WSN), which are synthesized under DC environments, are one of the proposed solutions that could be more efficient for energy savings. Recently, those systems have witnessed a large development, but they still struggle against competition in terms of efficiency and cost.

## DC power distribution system in PV-powered homes

Adopting and investigating DC-based power system in residential and commercial sectors has been implemented recently. The DC architectures have been investigated to motivate the consideration of such systems and their power quality. Several challenges compared with conventional AC-based distribution systems are also addressed.

Intel Corporation analyzed several power supply architectures and concluded that using a facility level of 400 V DC as a power distribution design could be the best choice to achieve end-to-end efficiency of 73% [1]. As indicated in [2], it is expected that an improvement of approximately 10% to 30% of system power efficiency is achieved by DC over AC. In AC-based power distribution, losses in power occur due to DC/AC and AC/DC conversions between the solar power source and the appliances that are already DC-internal appliances, while with DC-based systems, the power is delivered directly to the home load.

The major advantage of using a DC-based power system is the interconnection between system components with the back-up energy storage, which leads to minimizing the size of the generation process and the understanding of the DC-based power system [3]. A survey for DC-based power system quality and microgrids has been addressed [4]. To clarify the motivations of using DC power distribution with the household appliances, a brief classification can be followed as:-

1. Induction heater, as one of the largest electricity consumers among the household appliances, which considered to connect on DC supply system, the results show that the DC-bus allows the avoidance of the distortion of the additional current results in the capacitor of the DC-link and the induction load nonlinearity [5].
2. Resistive-based appliances; the household appliances can function directly on DC supply with full voltage range without any modifications [6–7].
3. Electronic appliances; where the complexity may appear when a semiconductor’s components become a part of the appliance power supply, but fortunately, the vast majority of the new household productions are designed such that they can be synthesized with a DC supply. Moreover, the operation of the appliance is dependent on the RMS value of the supply voltage with a range of approximately 200–300 V DC. Additionally, the incandescent light has a higher sensitivity to voltage variation and may decrease the lamp life. All electronic appliances can be operated on DC supply in the range of 100–300 V without any modification. The recommended value for supply voltage is 230 V DC, which is a suitable level if the AC electronics appliance is intended to operate on a DC source; this higher level in the voltage value is considered to reduce the consumed powered and the cost of the appliance [5].
4. Motor Driven Appliances; for appliances based on a universal motor, and when the DC voltage range is between 50 and 300 V, they achieve higher efficiency and long life time compared with the connection on AC [7–8].

## Traditional AC-based versus DC-based PV-powered and the proposed home system

**Fig 1:**
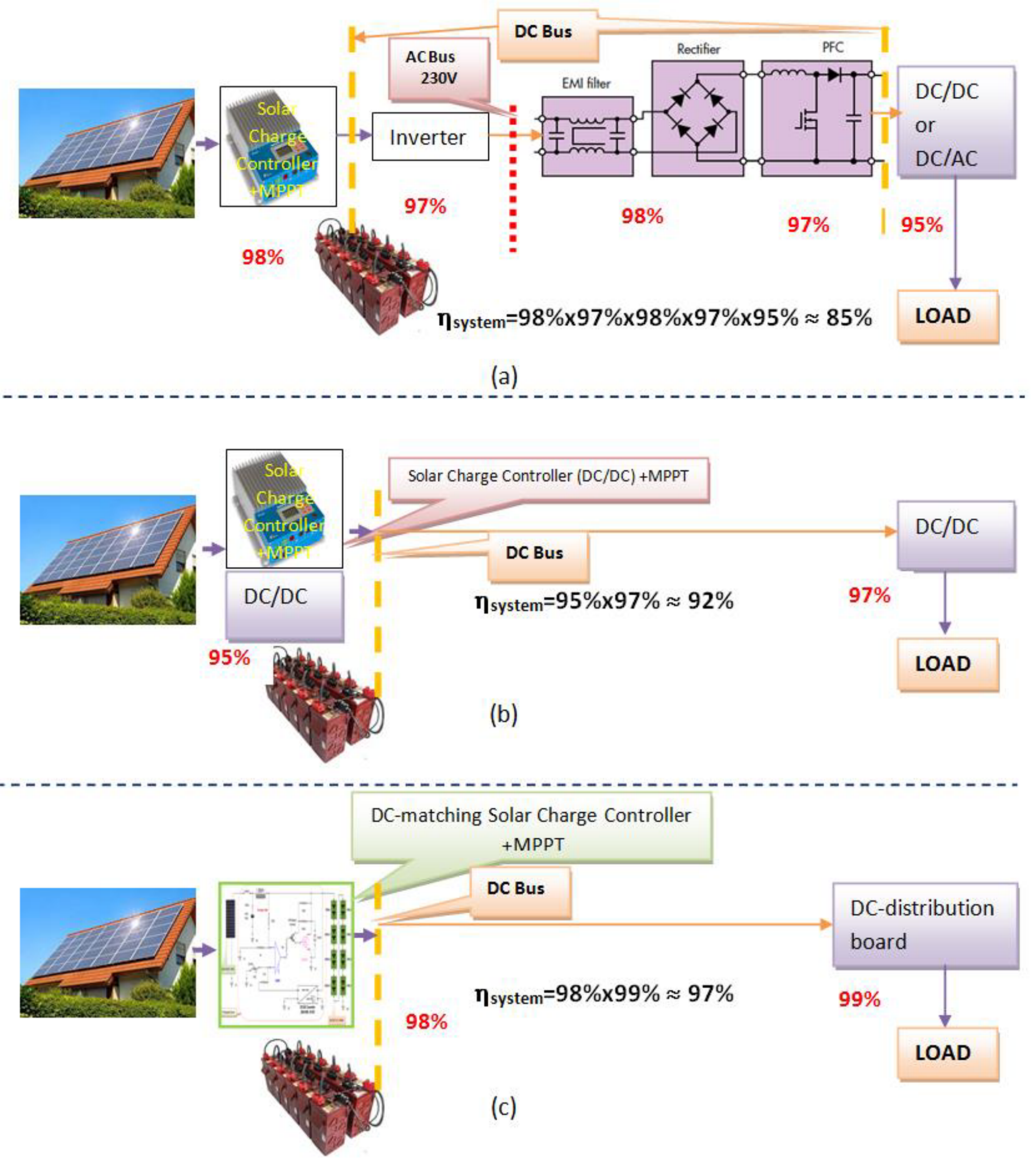
Solar-powered home system: Conceptual configuration. (a) Traditional AC-based, (b) DC-based, and (c) Proposed DC-based systems.

The configuration of AC-based Photovoltaic (PV) microgrid has been usually considered in the voltage range between (220–380) Vac. The maximum power point tracker (MPPT) manages a higher power from PV arrays to supply into an AC-bus or AC-grid through an inverter, as seen in Fig 1(A). For a typical household appliances application, such as personal computer, electronic devices, or variable speed motor-based appliance, they generally have a rectifier, an AC 50 Hz filter, a power factor corrector (PFC), and a DC/DC or DC/AC converter to deliver the loads with electric power. But, this AC-based system guides to a conversion loss with high amount. Therefore, the concept of “DC-based or DC-Bus PV” systems have been offered recently by our previous studies and others [9–11], the system configuration is demonstrated in Fig 1(B) and 1(C). The power conversion efficiency can be calculated in the AC-bus system, which is obviously less than that for the DC-bus system with about 12%. Besides, the DC-based PV system can also save a rectifier and a PFC circuits, the saving with the components cost about 25% [12]. Thus, the DC power distribution system is feasible in renewable energy applications.

An integrated design for a DC-based PV-powered home system must be explored and supported by practical impacts and features. This study addresses this application by means of various household appliances that may be considered for AC and DC environment systems.

## Smart homes

Real-time monitoring and energy management are major factors that play a role in smart homes. Thus, better ways to improve power consumption reduction are required. The development of power monitoring and display technologies have witnessed advancements in smart homes; the consumer becomes able to decrease the energy consumption or even to delay the demand of energy to reduce the electricity bill through the management of household appliance priorities while maintaining the current comfort level [13]. A design to function and examine an embedded smart home system, which incorporates energy storage resources applied in a smart home, is proposed by [14]. Intelligent hierarchical through the response of dynamic demand and energy resource distribution management act on the cost of the smart microgrid and can be very effective in falling the cost of energy consumption [15].

Recent studies and the new appliances’ productions indicate that smart homes will increase in number and create a more accessible and positively active green environment. Therefore, appliances and software companies have begun producing technologies and applications purposely built for smart home systems [16]. WSNs applied to monitor the consumption of energy for the household appliances that have been considered for use in tests, design and implementations [1]. WSN includes measurements for power consumption nodes with a central server, where the nodes check the appliance consumption, and send the measurements for more processing wirelessly to a central server. Different modules of wireless energy meters have been produced, utilizing the microcontroller together with current sensors or transducer circuits for energy management [17–19]. One study addresses a self-powered sensor module, as a micro-electro-mechanical system (MEMS), for data logging and measuring the instantaneous power [20]. This design connects some sensor modules to a passive proximity sensor and called on-board MEMS, but the attained energy from this process may not be sufficient to maintain the actuator function, such as the remote control of the ON/Off status.

The PV-powered smart home with electric vehicle energy storage and stochastic energy management also targeted by some studies to seek the charging energy cost over the peak-time of tariff and maintaining the variability of PV power [21].

## ZigBee-based communication in energy management of smart homes

Energy management for PV-powered residential building applications is a fast growing research aspect. The proplem of managing the power consumption rate can be formulated as a model predictive controller to achieve high performance (96–98)% for modeling and energy forcasting [22]. The three common network architectures of communication that are adopted in smart homes to monitor the performance of small-scale PV-powered systems are; ZigBee (250 kbps), Ethernet (10 Mbps), and WiFi (54 Mbps) as a wireless and wired technology. The significant factor was the cost-effective and competent network technology for monitoring and controlling such systems, and the reliability of data access was 98% [23].

To transfer the sensors’ data of energy measurements (such as: sensors, transducers and power meters) to the control and management devices vary with several features and the used communication technologies. These technologies can be classified into wire-based and wireless-based. Bluetooth devices, Wireless LAN, and ZigBee RF technologies have all been employed [17–20, 24]. As an example, some studies propose designs address fault detection and automatic control of a several main common electric appliances [17]. ZigBee-based wireless technologies characterize as a less expensive and simpler than other technologies, compete with Wi-Fi, in network areas [25–27]. A solid-state relay as an actuator is utilized to drive the low power signal generated by the ZigBee in energy management systems to turn off the power of the associated household appliance if it is overloaded [18]. An energy control system with new modification of power terminals to switch-off the power in the standby state is mentioned in [24]. A scheduling-based management system communicates the household appliances’ consumption with a smart meter, and storage units will lower the electric power cost of the consumer by reducing the expensive hours [28]. To highlight the difference between the two common wireless competitive technologies, Table 1 shows a survey comparison between the ZigBee and Wi-Fi properties.

**Table 1:** Comparison between ZigBee and Wi-Fi technologies.

	*ZigBee*	*Wi-Fi*
**Main Specification**	A high level communication protocols when utilized to design personal area networks built fro low-power and small size digital radios	Secure, reliable, high-speed internet and network communication wireless technology
**Based on**	IEEE 802.15.4 standard	IEEE 802.11. Generally, it operates in 2.4 GHz, 3.5GHz and 5 GHz.
**Data Rate**	Low data rate, and secure networking, suitable for periodic or intermittent data transmission from a sensor or input device	*Higher data rate*
**Power Consumption** [25, 29, 30]	Lower and long battery life (0.036 W)	Higher (0.210 W)
**Cost** [25–27]	Simpler and less expensive ($2.75–$3.5)	Higher cost ($3–$20)
**Network security**	Secured by 128 bit symmetric encryption keys	Secured by different encryptions
**In home automation applications**	Transmission distances range can be up to 100 meters depending on power output and environmental characteristics. Additionally, ZigBee-Pro can extend its ranges up to 1,600 m.	100 meters with data rates from 2 Mbps to 600 Mbps.
**The major drawbacks of the ZigBee technology [25]**	Small memory size, low processing capabilities, and can be interfered from other networks of same frequency band.	The power consumption and cost and of Wi-Fi is higher than other short-range wireless devices such as ZigBee.
**Field applications**	Wireless light switches, traffic management system, electrical power meters in home displays, and other industrial equipments that need short-range wireless data communications.	

**Fig 2:**
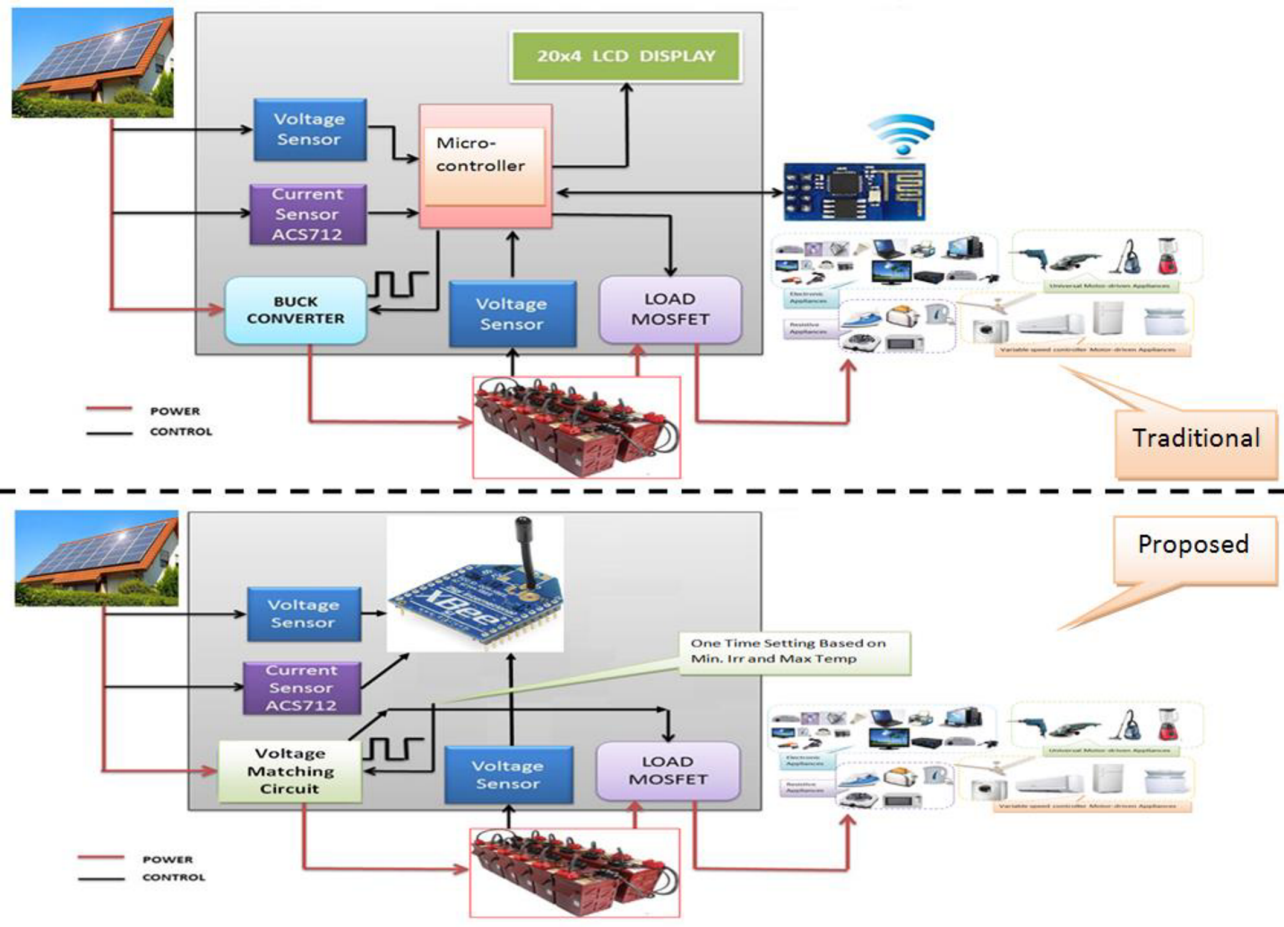
The difference between the traditional and the proposed system.

This work proposes an integrated wireless management system applied for a solar-powered house with a DC environment. The design includes a prototype that based on voltage matching concept between the source and load. Some of the important features that recognize the proposed framework are as follows:

1. A new control algorithm and circuit implementation for solar-battery charging process, which is based on utilizing the off-state energy at a high level of battery state of charge (SOC) period to be switched into another current path as an auxiliary load, furthermore, surplus power can deliver to a secondary battery or supply a ventilation fan for cooling the solar panels, which improves its performance. The control algorithm depends on a range of variation for both ambient temperature and irradiance geographically at the installation site, which is used to indicate the operational voltage of the system. The detailed algorithm presented in our previous work [31], which seems to be inversely designed, starts from the appropriate value of the load DC voltage to be assigned as the full charge voltage of the system’s batteries in the range (180–150)Vdc.
2. A solar-battery controller circuit. This circuit is designed to suit the DC-bus PV-powered home system with a suitable DC-DC power supply of a range (100–300) Vdc. The circuit and the system components adopt a single voltage (source-battery) matching concept to dispense the AC inverter. This part is described in detail in our previous work [7].
3. A new low power consumption WSN for energy monitoring which has a six precise analogue input channels to record the system measurements for both, the charging process, and energy parameters. The proposed wireless network consists of only a pair of XBee S1 Pro and depends on their capabilities for conducting the analogue to digital conversion (ADC) and for achieving the RF communication processes between the nodes.
4. The proposed approach is quite efficient in terms of user cost level contribution by adopting the concept of signal voltage matching, which adopts the suitability of system component selection, leading to the dispensing of additional microcontrollers and allows achieving a solid economic cost reduction. Finally, the above significant finding features can be described by Fig 2.

## Materials and methods

**Fig 3:**
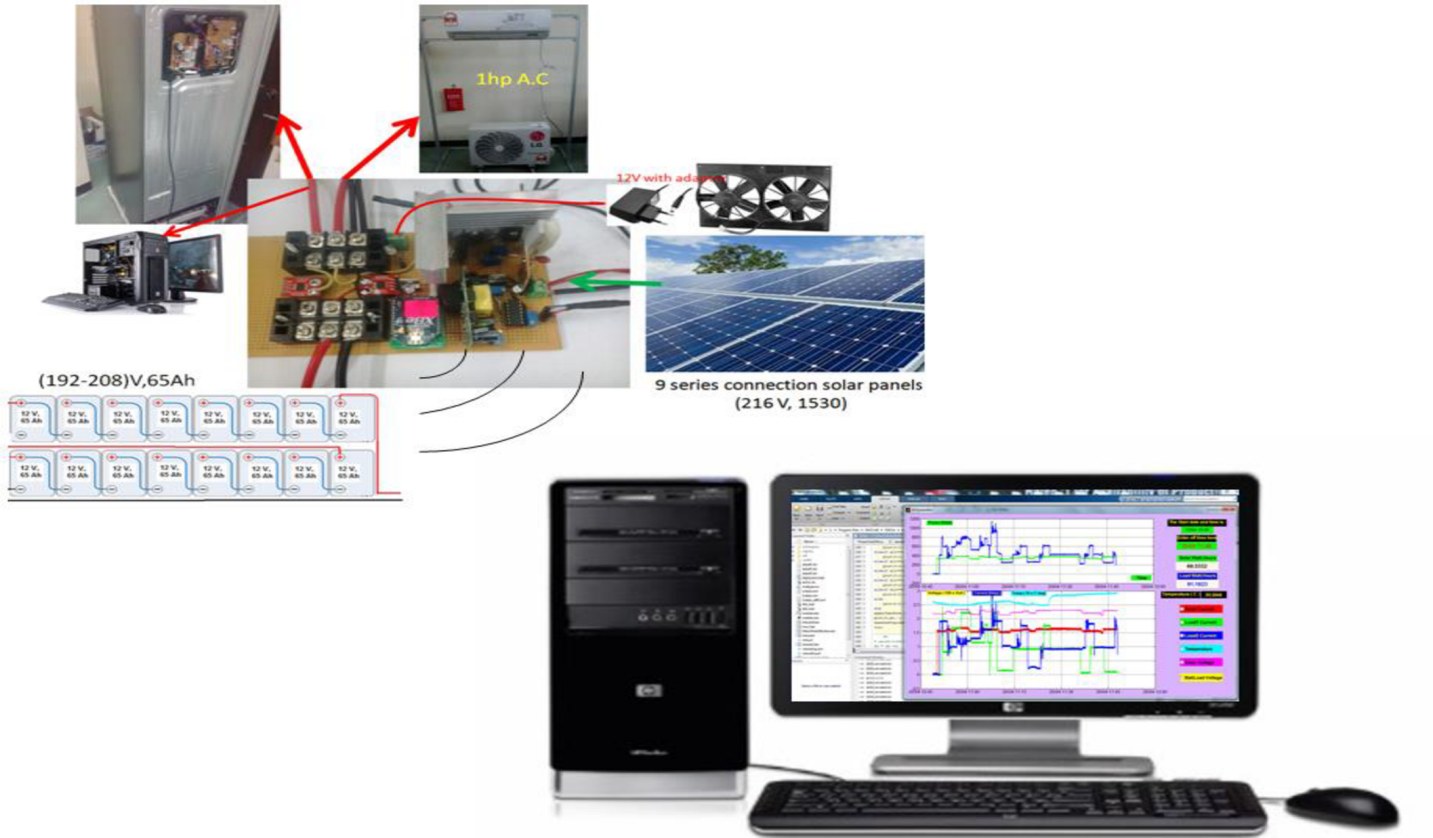
The diagram of proposed system.

The proposed framework consists of two parts: the hardware prototype module and the PC-based software. The prototype consists of a wireless energy management module and a solar-battery controller module, while the software can be divided into a graphical user interface (GUI) for hardware driving and data monitoring, and the initial configuration software. The proposed integrated prototype, which suits the functionalities required for the proposed system and the interconnection between system components, can be seen in Fig 3.

The prototype framework outcome provides measurements for monitoring the energy of the solar-battery-appliances chain. These measurements include parameters for two groups of home appliances and can be accessed through a GUI in real time with the ability for the user to recognize their power rate usage and adjust their actions to reduce the consumption. Fewer system components allow for energy monitoring economically of home management systems.

**Fig 4:**
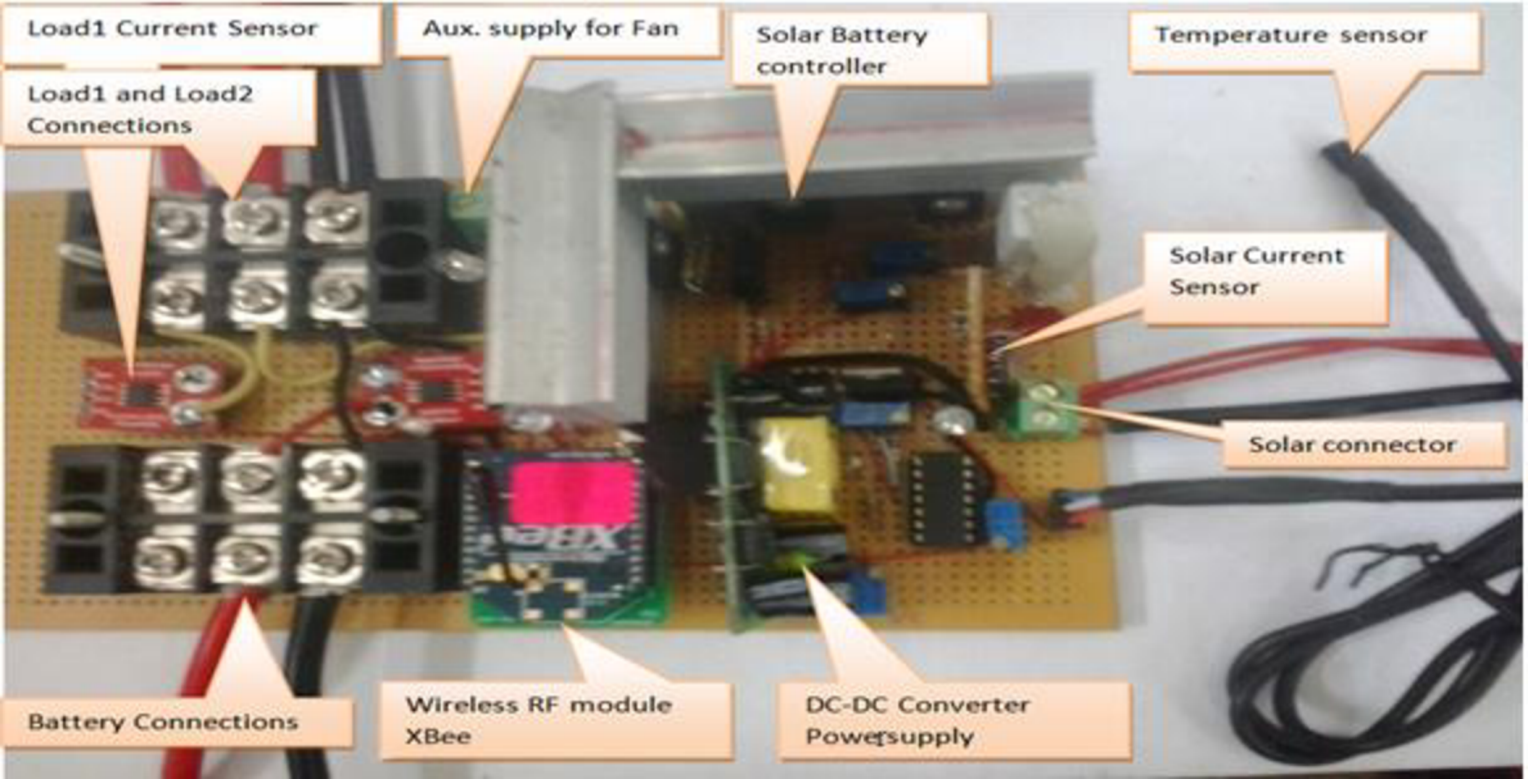
The proposed circuit board.

The testing of the proposed system relies on actual measurements from experiments that were performed on several local household appliances at University Putra Malaysia PV site. The solar array, batteries, and appliances were interconnected through the proposed prototype board, the prototype also includes DC-DC power supply, signal conditioning circuit, temperature, voltage and currents’ sensors, Those components accumulated in one board shown in Fig 4.

### Solar-battery controller module

**Fig 5:**
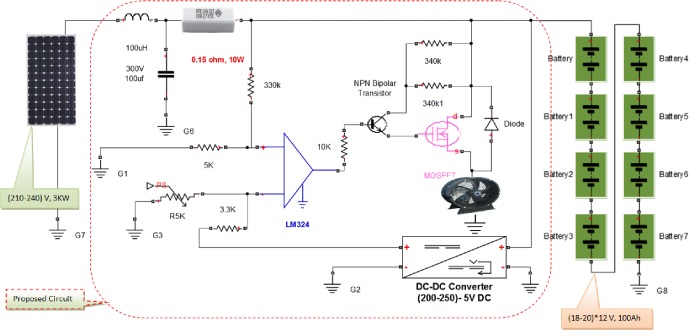
The proposed charge controller, one part of this system.

The main difference between the method adopted in this section of this work and that in previous related works, is that the power output of the solar PV array is linked directly to charge the battery bank at the interval in which the SOC of the battery bank voltage is at a lower level than its setting maximum value. The circuit acts to switch to another path at different interval in which the batteries reaches its full charge level, which is set previously according to the load compatibility with DC voltage supply. The switching process carried out through a MOSFET circuit to transfer the surplus power to an auxiliary load, such as fan or it can be used for a solar tracking. The schematic diagram that verifies this controller concept can be seen in Fig 5.

### Wireless energy management module

The proposed WSN for energy management consists of 2-nodes, each node has one XBee RF module, to process 6-analogue channels. The remote sensor node acts and configured as an end-device, while the other node is configured as a coordinator and acts as a central server attached to a PC or smart mobile phone. The 6-analogue processed channels can be classified as follows:

1. Two channels for voltage measurements (solar and battery/load voltages).
2. Three channels for current measurements (solar-battery, battery-load1, and battery-load2).
3. One channel with appropriate sensor for ambient temperature measurements.

**Fig 6:**
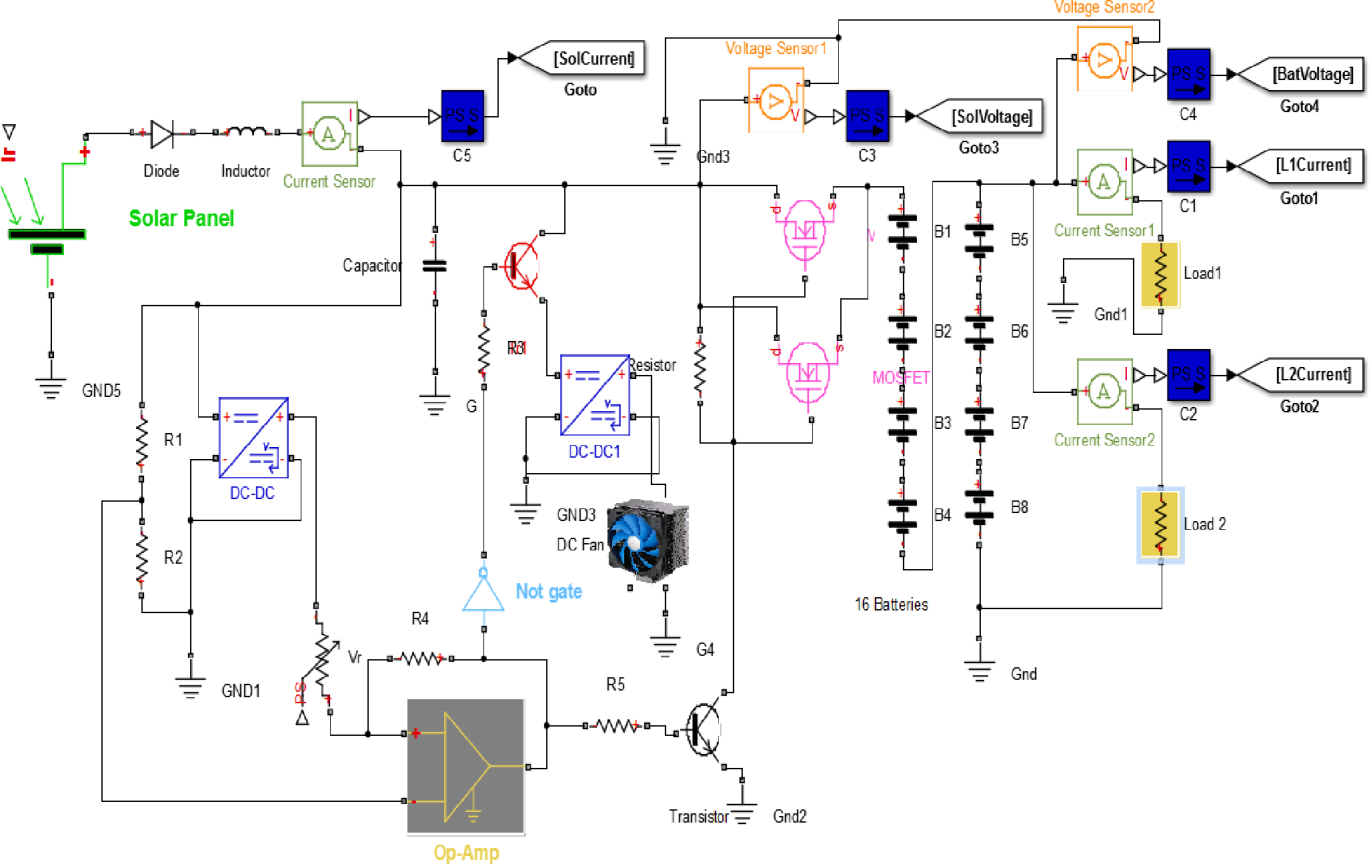
Solar-battery controller control proposed circuit with the energy sensors.

The circuit can be described as seen in the diagram in Fig 6.

The prototype power supply utilizes energy from the solar power source in the daytime, while using the backup battery of the system at night. The solar array and the battery power sources were connected to the load being monitored through the current and voltage sensors. The conditioner circuits of both current and voltage sensors designed by adopting LM324 op-amp, a Hall effect kit type (ACS712), were utilized as a current sensor, they were powered by 5 V from a DC-DC converter, 100–300 Vdc input and 5 Vdc output, which acts as a biasing power supply. The XBee RF module is powered with 3.3 V via a voltage regulator. The proposed circuit modified the application by synthesizing the connections between the sensors, and the direct connection with the XBee RF modules. Voltage measurements are achieved by using a resistive voltage divider circuit, the resistor values have been calculated so as to suit the minimization of power consumption and to limit the measured output values into lower than the limits of XBee analogue inputs. Each monitored household appliance is a DC-powered via a current sensor which allows the user to remotely manage the consumption of that appliance.

### Base station

**Fig 7:**
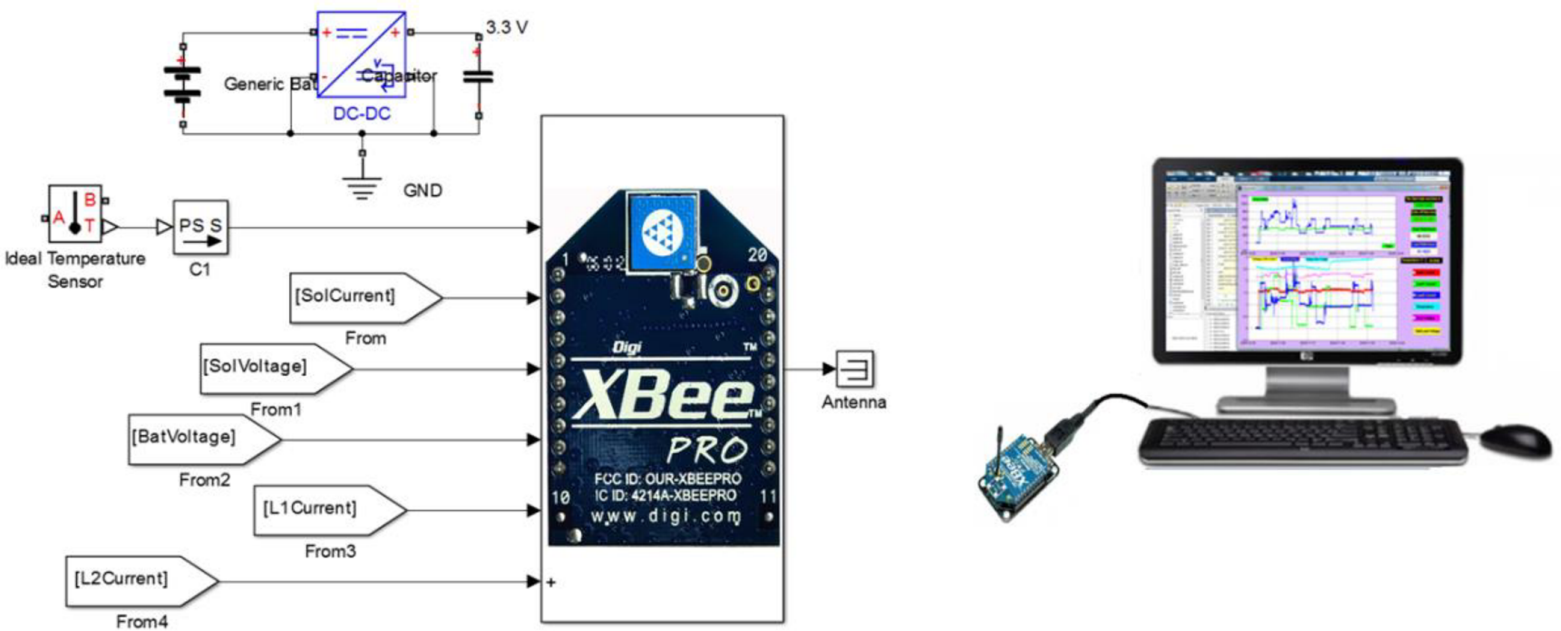
Wireless energy measurement and management module for the base station.

The base station is represented by the XBee coordinator module, the USB explorer board, and the central computer, and the module has been used to acquire the energy information from the node of remote sensors wirelessly. An FT232 chip has been used to provide a USB to UART interface, and MATLAB software was used to design the software driver for the remote configuration control of the remote sensor node. The GUI is used for displaying the attained data and monitoring the energy parameter measurements through the USB port. The simplest representation of the base station can be seen in Fig 7.

### Serial interface protocol

All sensors are synthesized and connected to the RF XBee module with IEEE 802.15.4 standard directly without any extra microcontroller card by the exploit of XBee capabilities. The XBee of the base station is configured to be operated under its application programming interface (API) mode, which is one of the two XBee’s modes to extend the interaction level between the remote host site and a module in the network. The API mode offers a structured interface for the data to communicate via serial line in structured packets. The process will assist in enabling the production of complex communications between the XBee module at the base station and that at end devices or remote modules without the need for protocol definitions [3].

**Fig 8:**
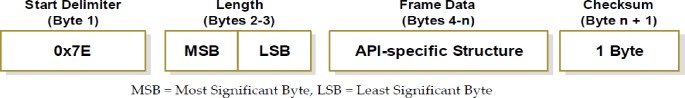
Frame structure of active API mode.

The communication starts with producing an API frame to expand the level at which the host function can cooperate with the networking capability of the XBee module. The serial, USB received data is queued up for the RF process to be transmitted. On active API mode (AP = 1), the frame structure of UART data is defined as shown in Fig 8.

The proposed work uses the maximum available six analogue channels for measuring and processing of temperature, currents and voltages that required for energy analysis, while using the digital I/O lines to control the appliances’ status.

### XBees’ initial configuration

Initially, the XBee Modules, at the base and remote nodes, need to be configured with the proper parameters according to the required ADC processes and to maintain data transmission between each other. In both XBee radios, XCTU is utilized to load the initial parameters, as listed in Table 2.

**Table 2:** Parameter configurations of the remote and base modules.

Parameter	Remote Configuration	Base Configuration
DL (Destination Address Low)	0x1234	0x5678
MY (Source Address)	0x5678	0x1234
AD = Analog‐to‐Digital Converter, DIO = Digital Input/Output	D0 = 2; D1 = 2; D2 = 2: D3 = 2; D4 = 2; D5 = 2	
IR (Sample Rate),	0x14	
P0 (PWM0 Configuration), P1 (PWM1 Configuration)		P0 = 2 P1 = 2
IU (Enable I/O Output)		IU = 1
IA (I/O Input Address)		0x5678 (or 0xFFFF)
API Operation (AP Parameter)		AP = 1

**Fig 9:**
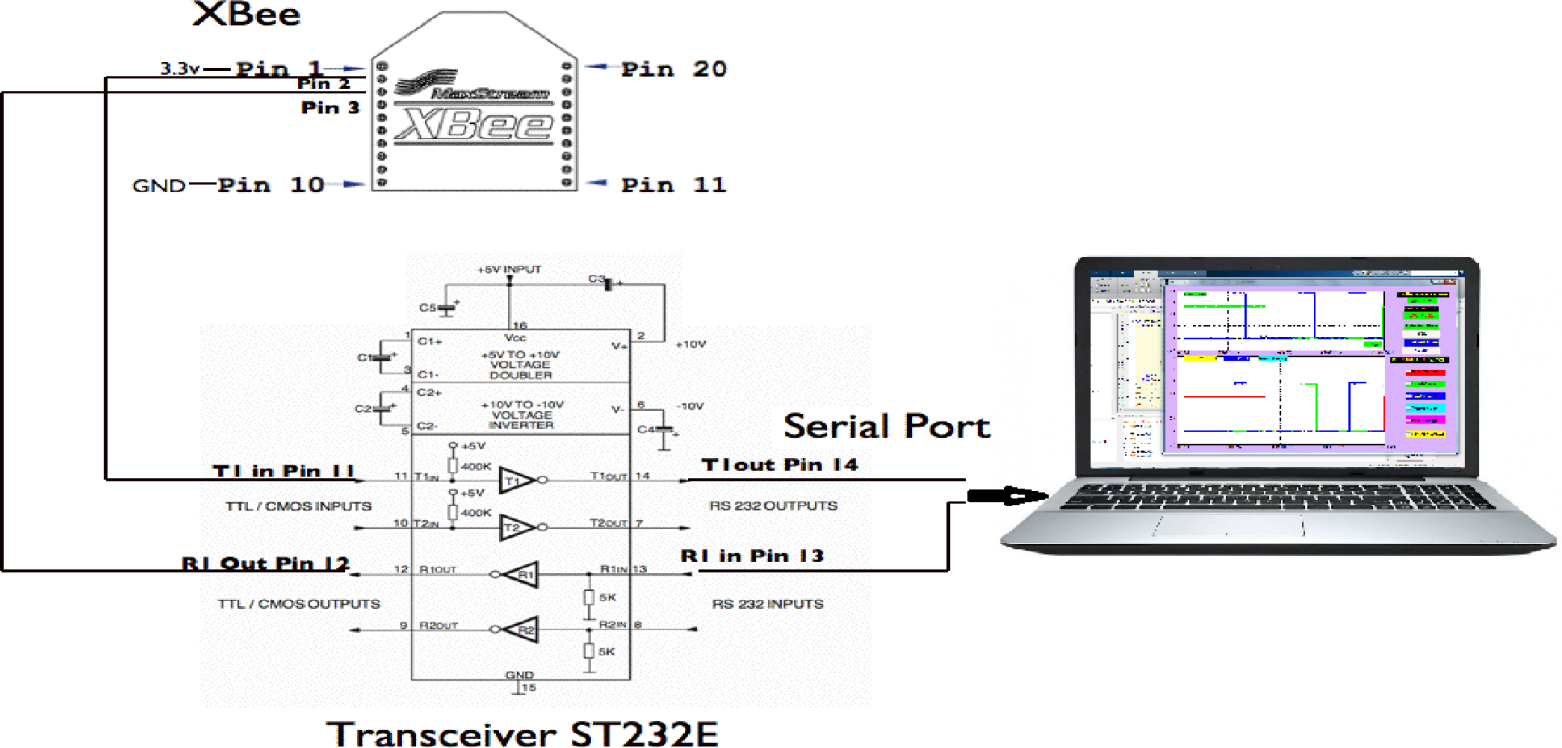
The 2 pin connection of the base Xbee and PC through transceiver.

The input/output ports of the remote RF module be configured as analogue inputs with a 20ms sampling rate, and the base/coordinator RF module, accordingly receives 24 Bytes (12 Bytes data and 12 Bytes framing) every 20ms. The hardware of the XBee base module includes only the connection between the base module and the monitoring PC via the transceiver chip ST232E, as shown in Fig 9.

**Fig 10:**
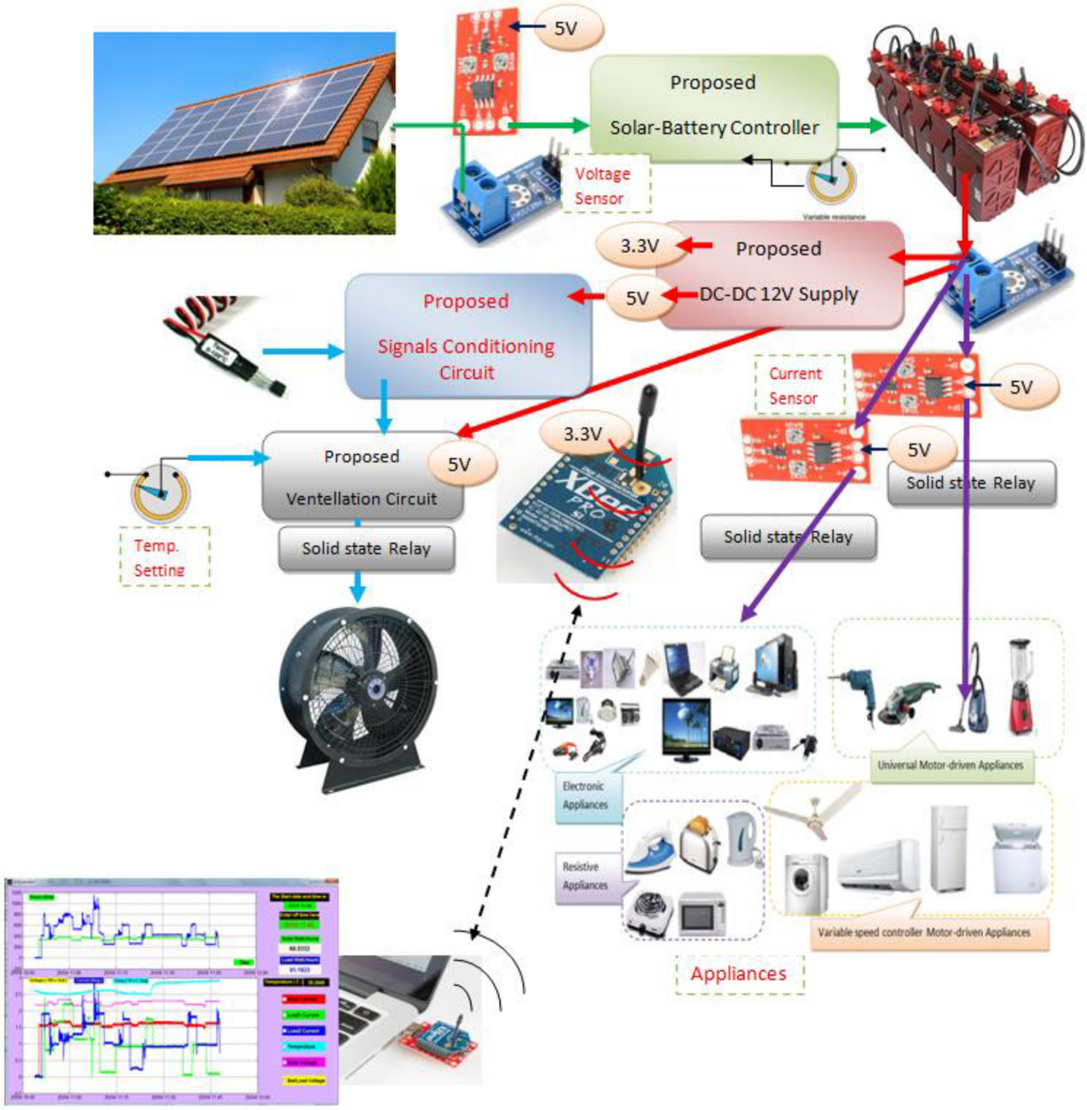
The proposed system diagram.

The components’ interconnection of the proposed system with their DC supply can be described as seen in Fig 10.

### PC software (GUI)

The GUI software has been designed for Windows environment and implemented using MATLAB code. The main purpose of the GUI is to monitor the power consumption rate of the household appliances, solar and battery bank energy measurements, and the ambient temperature in a real-time and to control the load status. The work’s MATLAB code is divided into two branches: Data accessing branch, and user input branch. The data transmission branch performed experimentally with a sampling rate of 11–123 Hz, at which the remote node transmits its data. The data accessing branch includes the following functions:

1. Creating a particular serial port, and Remote AT command.
2. Analyzing the received data, and checking the appliance status.
3. Saving of energy measurements in MATLAB workspace with maintaining the real-time update of GUI display.

**Fig 11:**
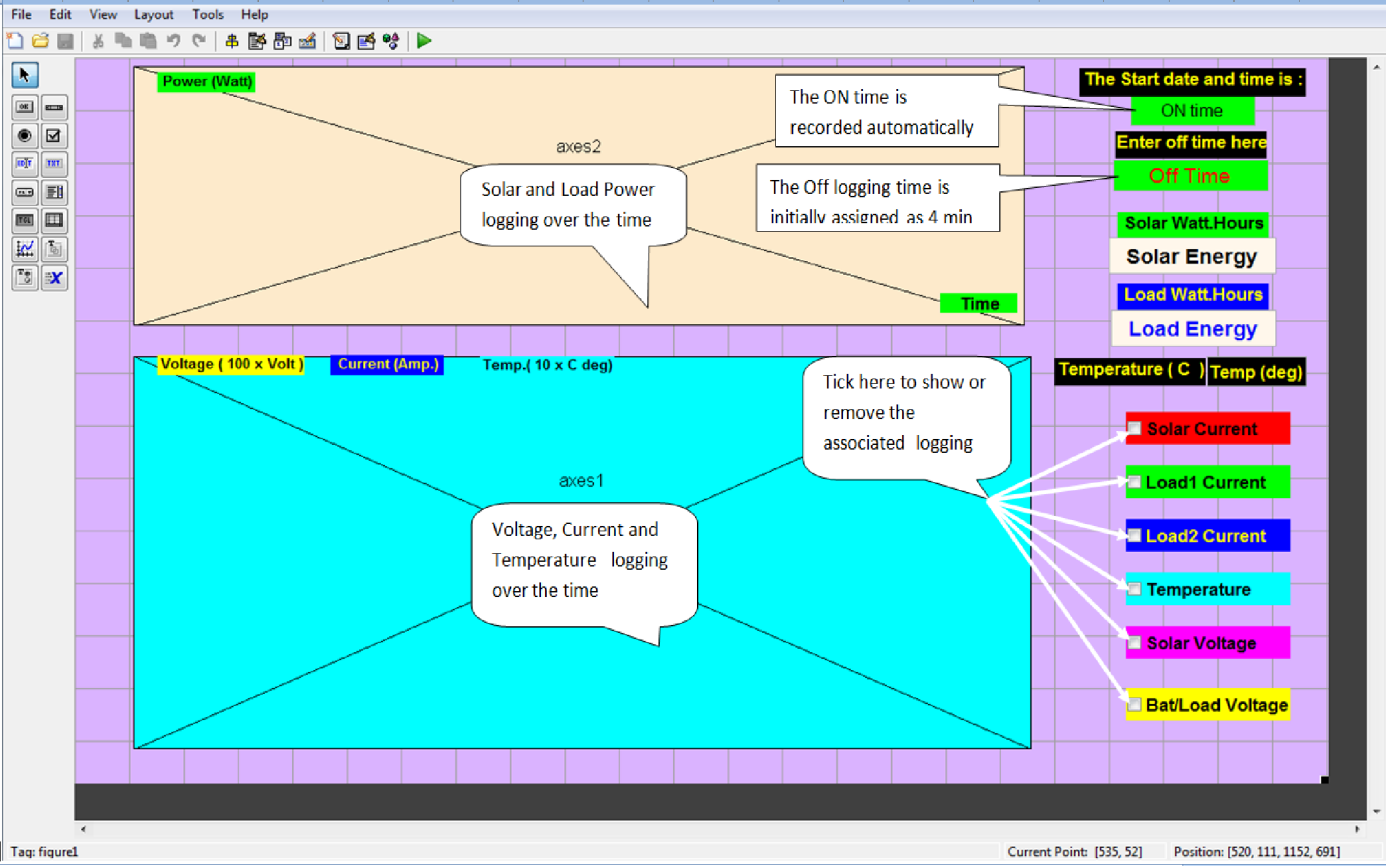
Proposed GUI for this work.

The designed GUI display contains Static Texts for naming and titles, Edit Texts for data input and real-time editing, Check Boxes for selecting between two options, and two X/Y axis display the appliances power consumption and status. The display always shows the on-line date and time while recording the measurements of energy parameters from the starting time to any stopping time. A 4-minute data logging has been assigned by default with flexibility to change the end of logging time (Fig 11).

**Fig 12:**
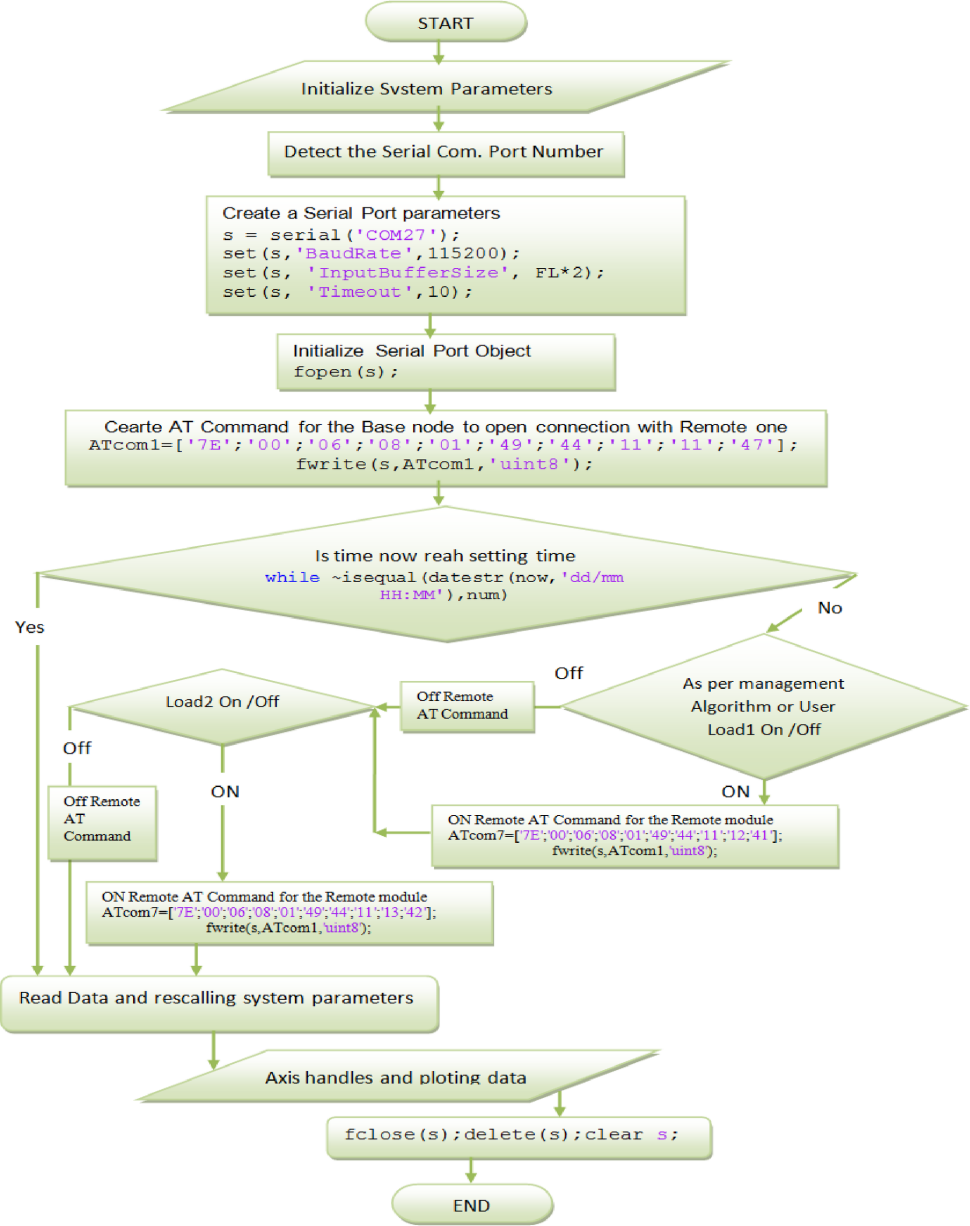
Flowchart of MATLAB GUI decoding.

The flow chart of MATLAB GUI Decoding is provided in Fig 12.

## Experimental results

Base and Remote Xbee RF modules create two types of data packets: eventual and periodic packets. The packet of the event is transferred occasionally as a response to the incident of a load status, while the periodic packet includes 24 bytes for the six channel measurements of voltages, currents and temperature. The sampling rate for the six analogue channels can reach up to 123 samples per second, which could be sufficient to capture the surge power of some appliances.

The proposed system has been applied on different types of household appliances, including the following:

1. Electronic appliances; PC with all accessories and printer.
2. Variable speed motor-based compressor load, which is also called Inverter Technology based appliances (refrigerator and air-conditioner).
3. Universal motor driven load, grinder, mixer.
4. Resistive load, water heater and electric oven.

**Fig 13:**
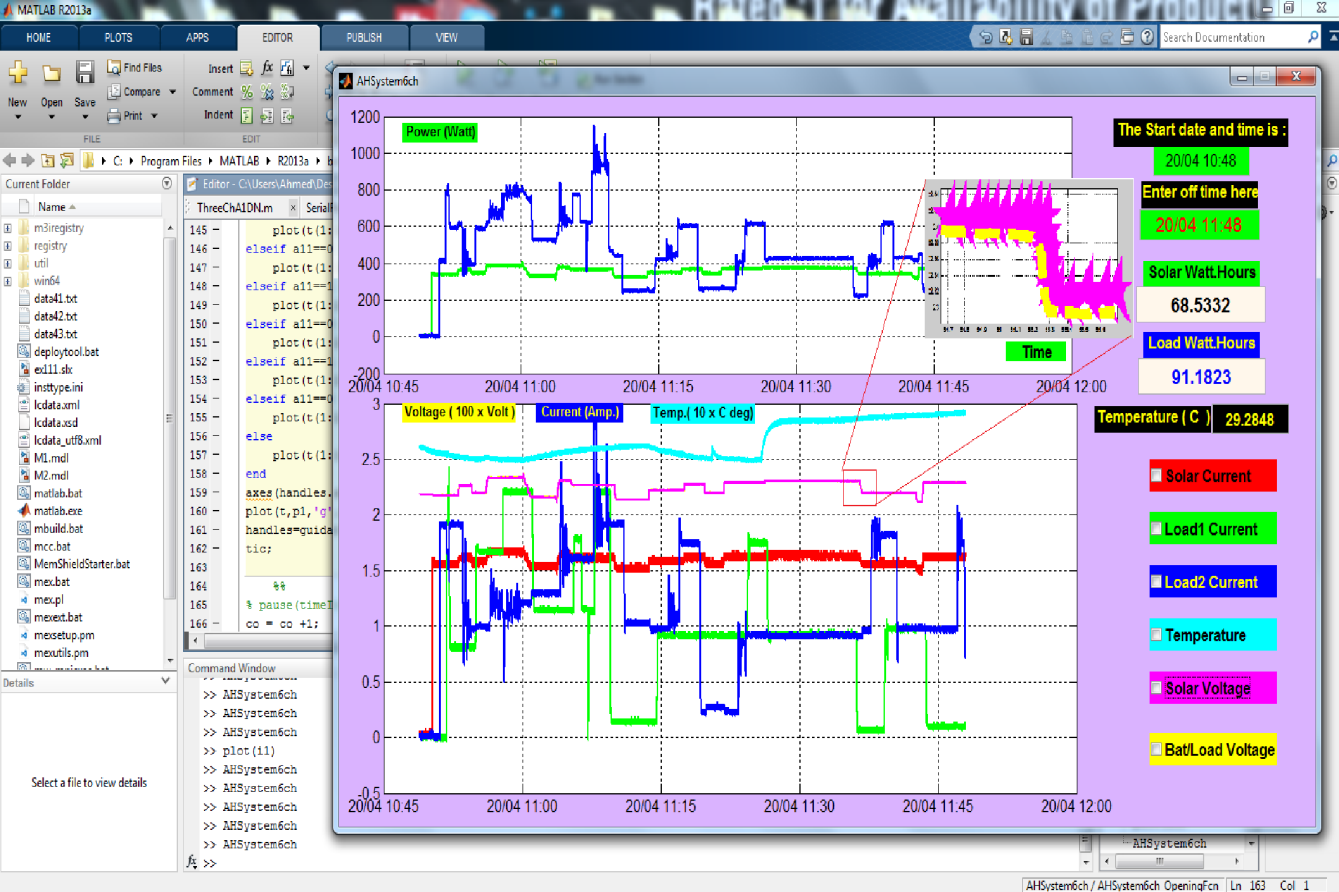
The variation of the values of all the energy consumption parameters.

Suitable scaling factors have been assigned to the ADC of the remote RF node. This is necessary because the measured values of voltage, current, and temperature need to be within the limit of 3.3 V, which is the reference voltage of the XBee. A sample of experimental real-time energy measurements for the proposed system was displayed over one hour data logging, can be seen in Fig 13.

It is clear that each parameter refers to its own color to display options of different load conditions to indicate whether the appliance is connected or not over the recording time. The indicated zoomed area on the GUI outcome proves the voltage matching between the solar array (as a source) and the battery/appliances (as a load), which represents a significant evidence for the considered matching concept.

### Research challenges

The challenges faced during the implementation of the work hypothesis can be seen in Table 3.

**Table 3:** Research challenges.

Issue Description	Proposed Solution and Components	Results
**DC source and AC appliances**	• DC voltage matching concept for all main system components. • Utilizing a Variable Speed BLDC motor driver for compressor based AC appliances and direct coupling for others.	• Very high efficiency. • System with minimum Power Converters.
**Minimizing of Losses**	• Dispensing of power converters based on the DC voltage matching concept. • Low frequency switching MOSFETs circuit	• Very low difference between the solar, battery and load voltages (Fig 13).
**Lower Cost**	• Using the appropriate components such as; Xbee module, Xbee USB driver base, 5 V DC adaptor, Lm324, MOSFET as a Solid-state relay, current sensor, voltage sensor, temperature sensor, vero-board and terminals and some electronic components.	• 128 USD
**Complexity**	• No additional microcontroller (such as Ardiuno, Raspberry Pi brand). • Matching concept already simplifies the circuits. • Suitable configuration of the XBee modules.	• Easy to understand
**Wireless Access**	• Six analogue channels, (11–123) sampling data rate • Direct Xbee coupling and XBee-based as a wireless sensor node only.	• Achieved

### Results evaluation

Three approaches can be considered within this topic: data processing and transmission, power calculations and the prototype efficiency, and the measurement performance.

#### Data processing and transmission

**Fig 14:**
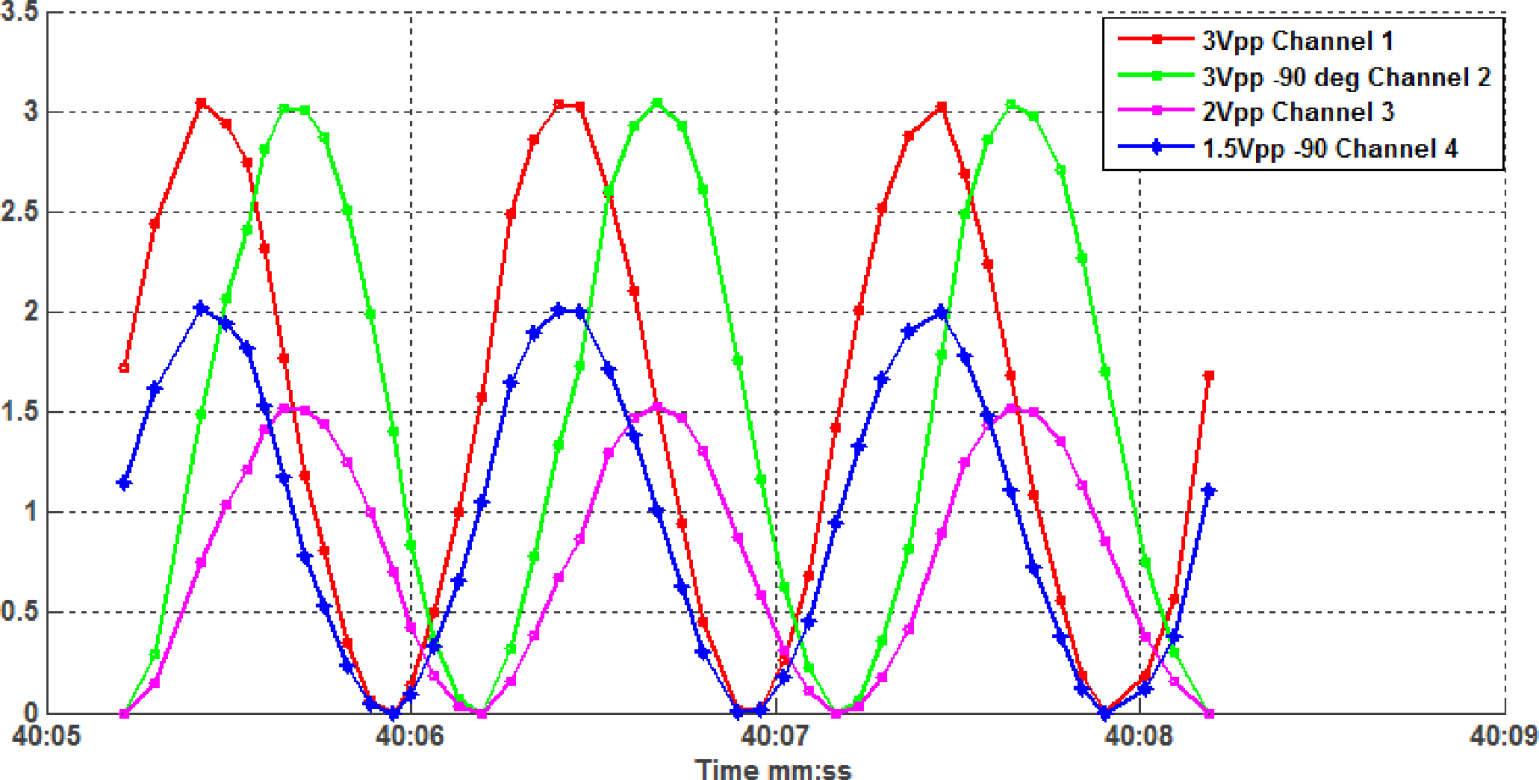
A four test signal received by the base station node that sent from the remote node wirelessly.

The proposed system can be feasibly verified by comparing test signals. Four sinusoidal waveform signals have been sent from the remote sensor node and have received by the coordinator RF module at a base station node. Sent and received signals can be seen in Fig 14.

The test signal has been sent with a peak value of 3, 3, 2, 1.5 volt respectively, with a -90 degree phase shift between each couple signals over 3 seconds time interval. The result shows accurate capturing for the sent data at about 14 sample/sec in this case.

The root mean square (RMS), which can be given by the standard Eq (1), is adopted to calculate the difference between the above four signals.

$$RMS\;error=\sqrt{\left(\frac{1}{n}\sum_{i=1}^{n}{\left(S_{si}-S_{ri}\right)}^{2}\right)}$$ (1)

where S_si_ and S_ri_, are the i_th_ sent and received signal respectively, and the RMS error was found to be in the range (0.01276–0.02867) for the 4 tested channels.

#### Power calculations and prototype efficiency

**Fig 15:**
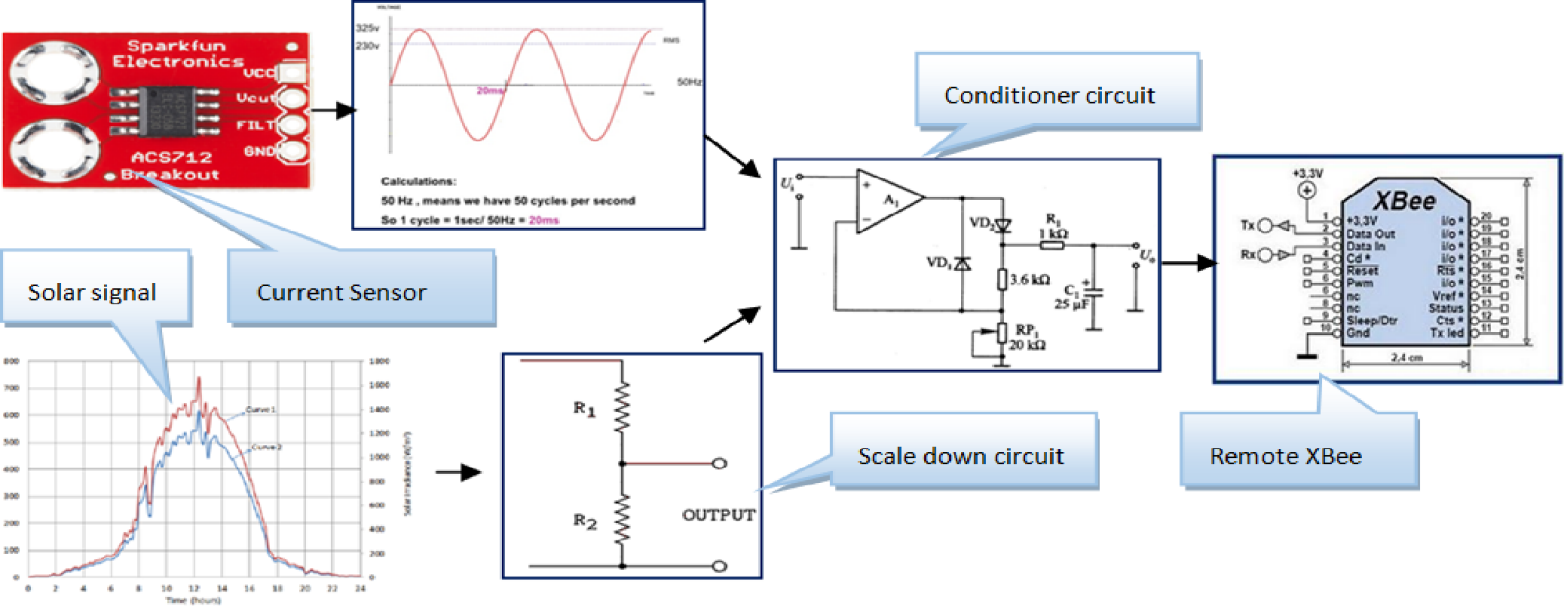
The measurement process for the currents and voltages of the proposed prototype.

As seen in Fig 15, the actual measured voltage value *V*_*t*_ has been calculated based on several signal conversion stages; therefore, the equation used to determine the voltages is given by:

$$V_{t}=K_{v}\;V_{out}=K_{v}\;\delta_{t}^{j}(V_{ref}/(2^{10}-1))$$ (2)

where V_out_ is the scaled down output voltage, which is limited by V_ref_ < V_out_ < 0. *K*_*v*_ is the scale down constant for the voltage signal and can be given by:

Kv=(R1+R2)/R2

where $\delta_{t}^{j}$, is the reading from channel (j) of the analog-to-digital converter at time interval (t).

Since the XBee S1 pro has a built in 10-bits ADC, therefore:

The step size is: 3.3 V/1024 = 3.22 mV. Similarly, for the current measurements,

$$\mathrm{Thus},\;I_{t}=V_{iout}\;K_{i}$$

where *V*_*iout*_ is the scaled down output voltage, which is equivalent to the measured current and limited by V_ref_ < *V*_*iout*_ < 0, while *K*_*i*_ is the current scaled down constant and can be given by

$$K_{i}=I_{max}/V_{ref}$$

$$I_{t}=K_{i}\;V_{iout}=K_{i}\;\delta_{t}^{k}(V_{ref}/(2^{10}-1))$$ (3)

The circuit uses V_ref_ = 5V with the current sensor model (ACS712ELCTR-05B-T);

Therefore, *K*_*i*_ *= 1*, and (3) becomes:

$$I_{t}=V_{iout}=\delta_{t}^{k}*(V_{ref}/(2^{10}-1))$$ (4)

where $\delta_{t}^{k}$, is the reading from the current channel *k*.

Thus, the total power of the a particular measured appliance can be calculated by using (5):

$$P_{t}=I_{t}*V_{t}$$ (5)

Where *P*_*t*_ represents the power of the measured appliance, while *I*_*t*_ and *V*_*t*_ denote to the current and voltage of that under measured appliance.

#### Measurement performance

**Fig 16:**
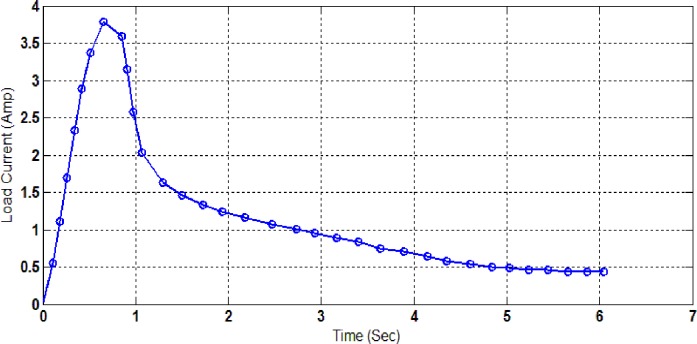
Proposed prototype performance by surge load current versus time.

One of the significant features in the proposed system measurements is the ability to monitor the surge current of a connected load, such as a motor-based machine at the start turning ON-time. To record such fast variation of the input analogue signal, a data rate of approximately 11 Hz has been set, this time, on 11 Hz to capture the surge in the measurement for monitoring purposes. An experimental test has been carried out on a mixer, which has a universal motor of 150 Watts rated power at no-load. The inrush current data have been monitored clearly when this appliance is connected to one of the two load current sensing channels of the proposed prototype (Fig 16).

**Fig 17:**
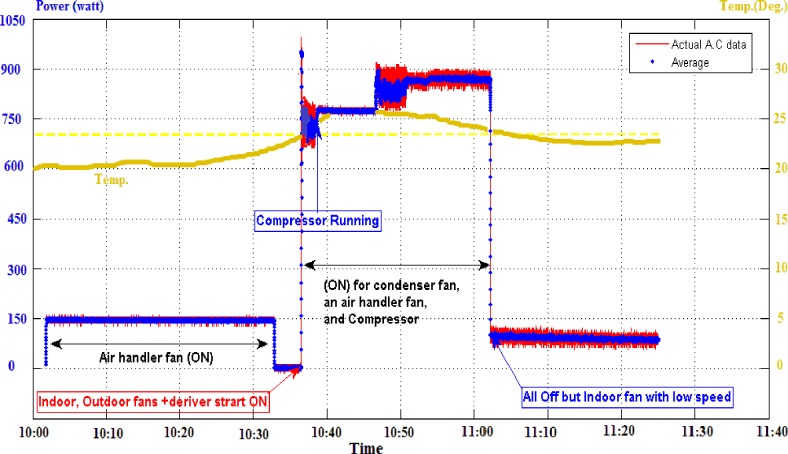
One and half hour period, experimental power consumption and temperature measurements from DC-bus supply on a 1 HP air-conditioner.

To inspect the system practically, an experimental measurements have been conducted on one of the most important consumer in homes which is the Air-Conditioner appliance. The main objective of this experiment is to evaluate the proposed prototype on a large energy consumer among household appliances for future further analysis and modeling (see S1 File). The power consumption rate data over one and a half hours have been recorded at different statuses from the air conditioner operation (Fig 17).

## Conclusions

A Source-Load Matching concept of a DC-based system together with WSN characterized by low-power-consumption has been proposed in this work. The work presents a framework for energy management suits the solar-powered home system in DC-power distribution system. An integrated prototype circuit is presented as a practical implementation to achieve the main work objective by focusing on the efficiency optimization in residential solar-power home systems. The hypothesis is based on the following premises:

- Based on the concept of source-load DC voltage matching, it may be possible to design a green-powered residential system with efficiency higher than that under the AC-based distribution system.
- Elimination of power converters will reduce the system losses.
- Using inverter technology in the recent productions of household appliances leads to motivate the use of DC-based microgrid environment, beyond any improvements that may be possible in an AC supply design.
- The zoomed area from the GUI outcome shows the matching in the voltage between the solar array and the battery/load, so that a consistent and logical interpretation of results is obtained.
- Only a pair of Xbee Pro RF modules with current, voltage and temperature sensors are sufficient for energy monitoring system.
- The implementation of the system design is recognized as an inexpensive method to monitor and manage household appliances’ consumptions via user awareness and interaction.
- The proposed GUI displays real-time measurements for two groups of household appliances, which can help consumers enhance their understanding of their energy usage patterns to adjust their actions to reduce costs and consumption.
- The proposed system has the flexibility to be expanded for unlimited remote sensing nodes, and for more general application within the DC-environment.

The obtained energy information can be then sent to a central remote server through the internet and makes the proposed system an economical, time- and labor-saving solution for large scale power system applications.

## Supporting information

S1 File Video shows the GUI of the real-time measurements of the proposed system. (MP4)
